# Downregulation of the Sonic Hedgehog/Gli pathway transcriptional target Neogenin-1 is associated with basal cell carcinoma aggressiveness

**DOI:** 10.18632/oncotarget.21061

**Published:** 2017-09-19

**Authors:** Bárbara S. Casas, Christelle Adolphe, Pablo Lois, Nelson Navarrete, Natalia Solís, Eva Bustamante, Patricio Gac, Patricio Cabané, Ivan Gallegos, Brandon J. Wainwright, Verónica Palma

**Affiliations:** ^1^ Laboratory of Stem Cells and Developmental Biology, Faculty of Sciences, Universidad de Chile, Santiago, Chile; ^2^ Institute for Molecular Bioscience, The University of Queensland, Brisbane, Australia; ^3^ Faculty of Medicine, Universidad de Chile, Santiago, Chile; ^4^ Fundación Arturo López Pérez, Santiago, Chile; ^5^ Universidad de Chile Clinical Hospital, Santiago, Chile

**Keywords:** BCC, Neogenin-1, SHH/GLI pathway, Netrin-1, tumor aggressiveness

## Abstract

Basal Cell Carcinoma (BCC) is one of the most diagnosed cancers worldwide. It develops due to an unrestrained Sonic Hedgehog (SHH) signaling activity in basal cells of the skin. Certain subtypes of BCC are more aggressive than others, although the molecular basis of this phenomenon remains unknown. We have previously reported that Neogenin-1 (NEO1) is a downstream target gene of the SHH/GLI pathway in neural tissue. Given that SHH participates in epidermal homeostasis, here we analyzed the epidermal expression of NEO1 in order to identify whether it plays a role in adult epidermis or BCC. We describe the mRNA and protein expression profile of NEO1 and its ligands (Netrin-1 and RGMA) in human and mouse control epidermis and in a broad range of human BCCs. We identify in human BCC a significant positive correlation in the levels of *NEO1* receptor, *NTN-1* and *RGMA* ligands with respect to *GLI1*, the main target gene of the canonical SHH pathway. Moreover, we show via cyclopamine inhibition of the SHH/GLI pathway of *ex vivo* cultures that NEO1 likely functions as a downstream target of SHH/GLI signaling in the skin. We also show how *Neo1* expression decreases throughout BCC progression in the K14-Cre:Ptch1^lox/lox^ mouse model and that aggressive subtypes of human BCC exhibit lower levels of NEO1 than non-aggressive BCC samples. Taken together, these data suggest that NEO1 is a SHH/GLI target in epidermis. We propose that NEO1 may be important in tumor onset and is then down-regulated in advanced BCC or aggressive subtypes.

## INTRODUCTION

According to the International Agency for Research on Cancer (IARC, WHO) basal cell carcinoma of the skin (BCC) is one of the most common neoplasms worldwide, with a 10% increase in incidence every year. UV radiation is one of the main risk factors for the acquisition of BCC, therefore it develops most frequently in areas of the skin that are most exposed to the sun and its incidence increases with age [1, 2].

Clinically, BCC is a collection of slow growing, telangiectatic nodules with epidermal basal cell resemblance. However, there is a lot of histo-morphological variability among BCCs which exhibits distinct gene expression patterns [3]. Incomplete excision, frequent occurrence, increased risk of subclinical spread and aggressive local behavior are characteristics of high-risk aggressive BCC, consisting of micronodular, infiltrative and sclerosing BCC subtypes. In contrast, nodular BCC (comprising 60–80% of BCCs) and superficial BCC (comprising 10% of BCCs) exhibit a slow growing and non-invasive growth pattern, these tumors form cellular nodules that do not infiltrate the dermis and are therefore easier to resect [4–6]. In the large majority of cases, BCC has been found to develop due to constitutive activation of the Sonic Hedgehog/GLI (SHH/GLI) pathway. Typically, SHH binds to its receptor, Patched1 (PTCH1), which results in loss of Smoothened (SMO) repression. SMO is subsequently phosphorylated which results in the accumulation of transcriptional GLI activators that drive the expression of hedgehog target genes such as *PTCH1, GLI1,* and *BCL2,* among others [7, 8]. In the skin, SHH/GLI signaling regulates hair follicle growth and morphogenesis, allowing the initiation of anagen (growth) phase, where the expression of SHH and the ability of cells to respond to this signaling is temporally and spatially regulated during the hair follicle cycle [9, 10].

In human BCC, constitutive activity of the SHH/GLI pathway is most commonly attributed to inactivating mutations in *PTCH1*, resulting in unrestricted SMO activity [11, 12]. Moreover, heterozygous deletion of *PTCH1* occurs in Gorlin syndrome, a disorder that predisposes patients to a wide range of tumours, including development of BCC [13–15]. Although the majority of BCCs have been shown to exhibit increased SHH/GLI pathway activity, each subtype exhibits a unique gene expression profile [3]. To date, it remains unclear as to whether aggressive tumor growth is attributable to events downstream of SHH/GLI pathway activation or to other yet unidentified pathways.

Neogenin-1 (NEO1) is a receptor that was recently reported as a transcriptional target of the SHH/GLI pathway in the central nervous system [16]. NEO1 has two main ligands, Netrin-1 (NTN1) and RGMA, which are chemotactic molecules for axonal guidance during neural development [17]. NEO1 has also been proposed to be a death dependence receptor (DDR) [18, 19]. In presence of their ligands, DDRs promote positive signaling, such as cell proliferation, migration and survival, and in absence of their ligands, they can lead to apoptosis, therefore they are proposed to be involved in tumorigenic processes [20]. NEO1 has been reported to be deregulated in several cancer types but little is known about its specific function in cancer cells. In many of these cases, the deregulation of NEO1 is associated with elevated tumor aggressiveness and progression [21–25].

In the present study, we show that NEO1 is expressed in both human and mouse skin and that its expression decreases as murine BCCs develop in the K14Cre:*Ptch1^lox/lox^* mouse model [26]. While non- aggressive human BCC subtypes display high NEO1 expression, aggressive human BCC subtypes present with lower levels of NEO1, similar to *GLI1*, suggesting a possible role of SHH/GLI/NEO1 signaling in tumor aggressiveness.

## RESULTS

### NEO1 is expressed in the proliferative basal cell compartment of the skin

**Figure 1:**
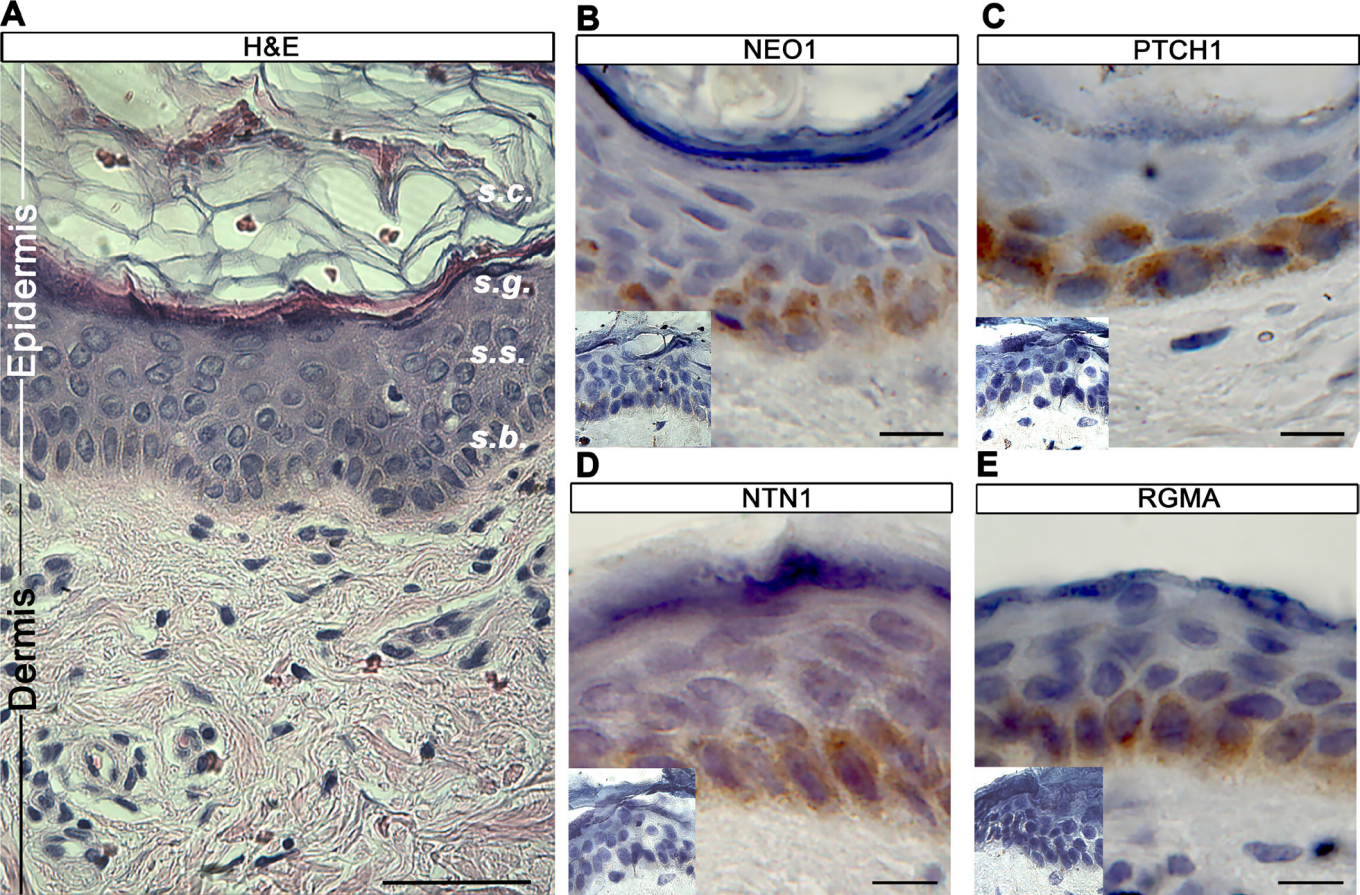
NEO1 is expressed in human epidermal basal cells (**A**) H&E of human skin showing stratum corneum *(s.c),* granulosum *(s.g.)* squamous *(s.s.),* and basale (s.b) of epidermis (scale bar = 250 μm). IHC analysis of NEO1 (**B**), PTCH1 (**C**), NTN1 (**D**) and RGMA (**E**) shows expression (brown staining) in the basal cell population of human skin epidermis. Hematoxylin (blue) counterstain used to distinguish nuclei. All images are representative photographs from *n* = 3 (bar = 25 μm). Negative controls are shown as insets.

Although NEO1 is detected in human skin, as indicated by Genome-wide transcriptomics and proteomics [27], to date a detailed study on its expression pattern is not available. Therefore, we first decided to examine the expression of NEO1 in control (not cancerous) human skin via immunohistochemistry (IHC) staining in order to describe its histological distribution and to correlate its expression levels with SHH/GLI pathway activity. Human epidermis is a polystratified epithelium composed of several layers of flattened (squamous) epithelial cells, acting as a tight water-proof barrier (Figure 1A). We observed NEO1 protein expression was restricted to the *stratum basale* (*s.b*) or basal layer, directly adjacent to the dermis (Figure 1B), which is where epidermal progenitors reside. This is the same epidermal compartment in which PTCH1 expression is observed (Figure 1C). Hence NEO1 is expressed in the same cell population responsive to SHH/GLI signaling, these data are consistent with NEO1 being a downstream target of that pathway. We subsequently set out to address whether NEO1 ligands were expressed in control epidermis and observed the two main NEO1 ligands, NTN1 and RGMA, were expressed in the basal layer (Figure 1D and 1E). Expression of NEO1 ligands in the same cell population as NEO1 itself is consistent with NEO1 acting as a dependence receptor.

**Figure 2:**
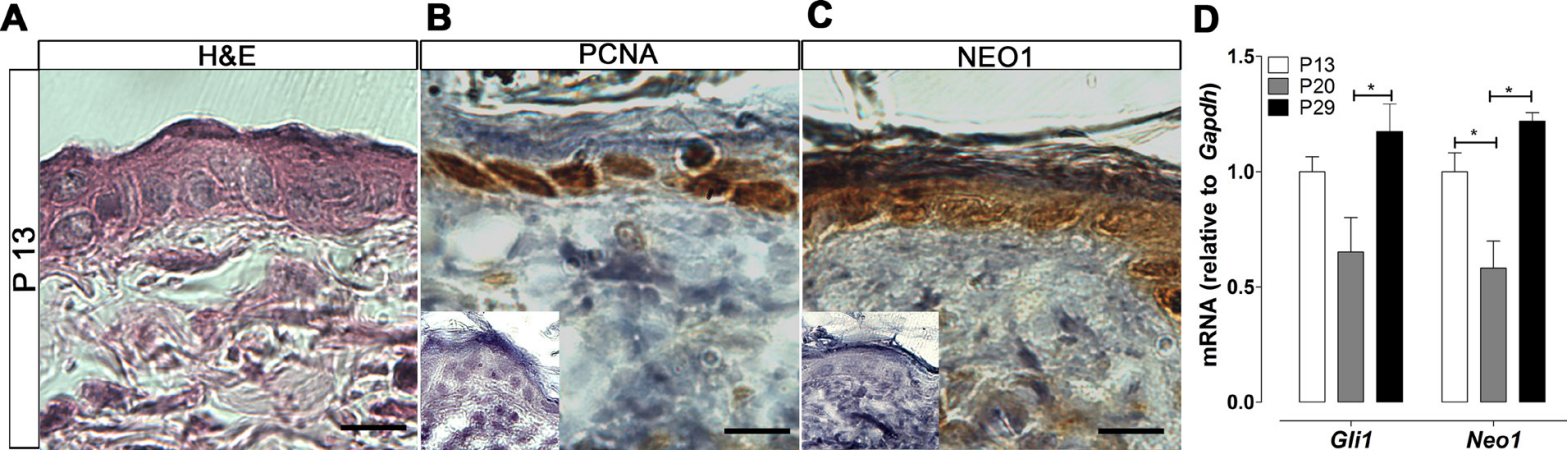
Neo1 expression and Shh/Gli pathway activity cycle in post-natal mouse skin (**A**) H&E of mouse epidermis. IHC analysis of the proliferation marker PCNA (**B**) and NEO1 (**C**) shows expression (brown stain) in the proliferative basal cell population. Images are representative photographs of *n* = 3 on P13 (bar = 25 μm). Negative controls are shown as insets. (**D**) mRNA levels of both *Gli1* and *Neo1* were assessed by qPCR, the expression of *Gli1* and *Neo1* cycle across different ages (*n* = 8). Data is shown as Mean ± SEM and normalized against mean value for P13 with *p < 0.05 according to Mann-Whitney test.

We next assessed NEO1 expression in mouse skin, which has an identical stratified structure as human skin, albeit thinner (Figure 2A). Indistinguishable from human skin, murine NEO1 protein is restricted to the proliferative (Figure 2B) epidermal basal layer (Figure 2C and Supplementary Figure 1). The level of SHH/GLI pathway activity is variable during postnatal epidermis, namely attributable to changes in the hair follicle cycle. Pathway activity is higher during periods of hair follicle growth (anagen) and lower during the phases of hair follicle regression and rest (catagen and telogen respectively) [9]. As expected, we observed highest levels of *Gli1* mRNA (used as a read-out of Shh/Gli pathway activity) at time points that correlate with stages of HF growth (P13 and P29) (Figure 2D). Not only did the level of *Neo1* mRNA follow the same cyclical trend as *Gli1*, moreover, the level of *Neo1* was similar to the level of *Gli1* at all three ages analyzed. In contrast, the expression level of Neo1 ligand mRNA was much lower (*Ntn1* is almost undetectable, *Rgma* is about 10-fold lower); they also fail to cycle along HF stages (Supplementary Figure 2). Analogous to Neo1 being a neuronal target of Shh/Gli pathway activity, these data support Neo1 as a potential downstream target of Shh/Gli1 pathway activity in the skin.

### NEO1 expression in BCC

**Figure 3:**
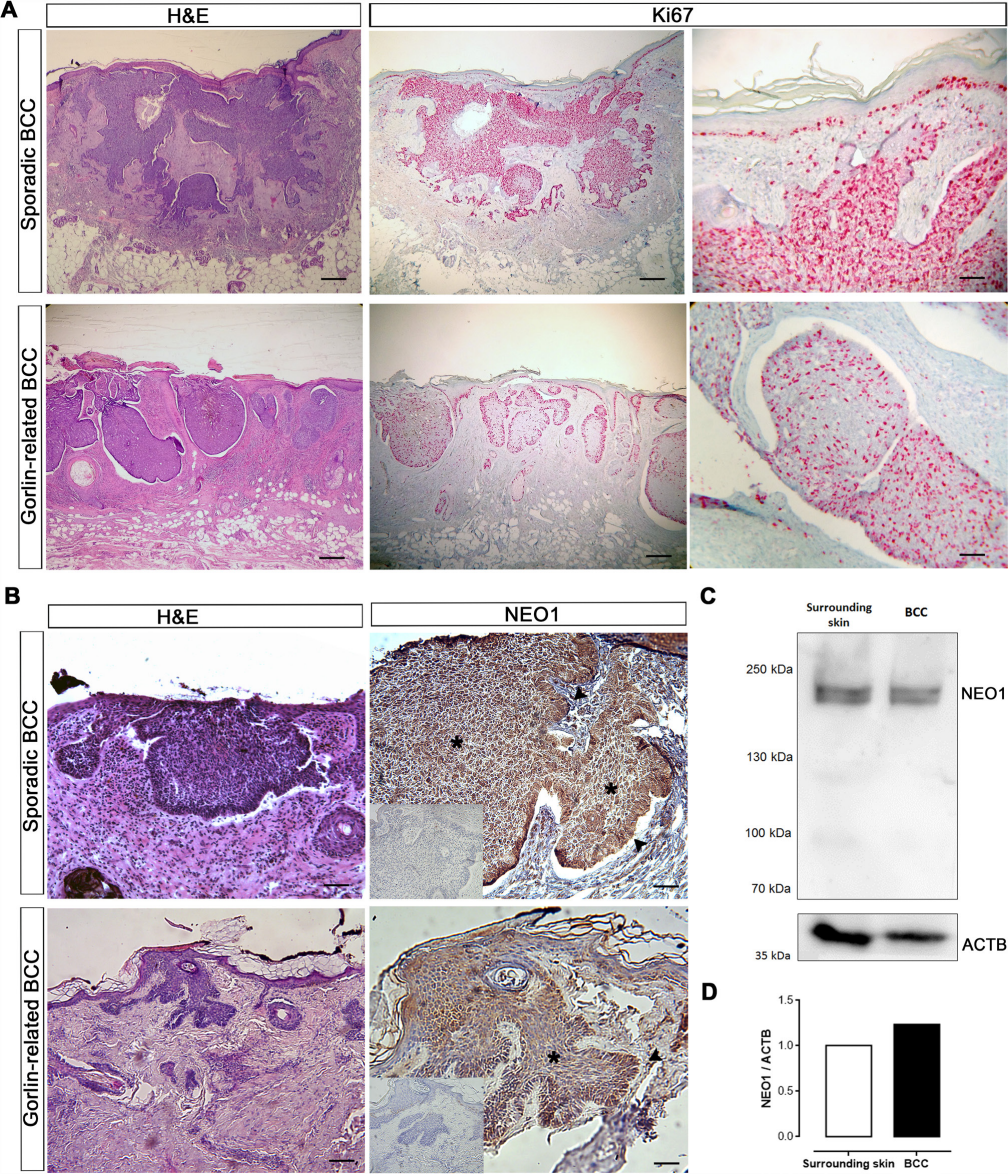
NEO1 is expressed in human BCC (**A**) H&E of sporadic and Gorlin syndrome-related BCC biopsies (bar = 1000 μm). IHC of Ki67 (pink stain) shows highly proliferative tumor cells (bar = 1000 μm and 250 μm). Images are representative photographs of *n* = 32. (**B**) H&E of sporadic and Gorlin syndrome-related BCC biopsies (bar = 1000 μm). IHC analysis of NEO1 shows expression (brown staining) in nodules of human BCC biopsies both in the bulk of tumors (asterisk) and palisade (arrow). Hematoxylin (blue) counterstain was used to distinguish nuclei. Negative control of IHC is shown as an insert. Images are representative photographs from *n* = 32 for sporadic BCC and *n* = 4 for Gorlin syndrome-related BCC (bar = 500 μm). (**C**) NEO1 expression in a non-aggressive sporadic human BCC and its healthy surrounding skin was evaluated by WB, ACTB is shown as a loading control. (**D**) Graph depicting the levels of NEO1 in BCC compared to its surrounding skin evaluated in (c) and normalized by ACTB expression.

We next set out to address whether NEO1 was expressed in BCC. BCC is often characterized as a hyperproliferative mass of basal cells. We confirmed that our cohort of sporadic and Gorlin-related BCC was indeed highly proliferative (Ki67 immunoreactivity) (Figure 3A). In order to determine the spatial distribution of NEO1 within the neoplasm, we screened both sporadic and Gorlin-related BCCs by IHC. In sporadic BCC, we observed that NEO1 was uniformly expressed throughout the tumor lesions. Consistent with its cellular distribution in control epidermal basal cells, we observed nuclear NEO1 expression within the bulk of the tumor (Figure 3B asterisk). In the palisading basal cells, were high GLI1 expression has been described before [12], the staining for NEO1 was stronger and diffused within the cytoplasm (Figure 3B arrow). Even though, NEO1 staining was stronger in the palisading cells of Gorlin-related BCC, similar to the staining in sporadic BCC, the overall staining of NEO1 was more diffused in the bulk of the tumor (Figure 3B asterisk) with a more cytoplasmic distribution. Comparison of IHCs of control skin (Figure 1B) and BCC (Figure 3B) reveal that NEO1 is highly expressed in tumor sections compared to control skin. To corroborate the latter, we evaluated NEO1 protein abundance through Western blotting (WB) in whole BCC tumor tissue extracts and compared it with whole skin extracts from adjacent region (Figure 3C). We confirmed that there is a higher NEO1 expression in BCC compared to its surrounding healthy skin (Figure 3D). In addition, we assessed the protein abundance of NEO1 and ligands in a small cohort of sporadic human BCC tumor samples by WB. We detected varying levels of NEO1 among the samples. Interestingly, NTN1 was detected in all the samples analyzed, while RGMA was detectable in only 3 of them (Supplementary Figure 3). Of note, we also observed NEO1 expression in eccrine sweat glands and sebaceous glands (Supplementary Figure 4).

**Figure 4:**
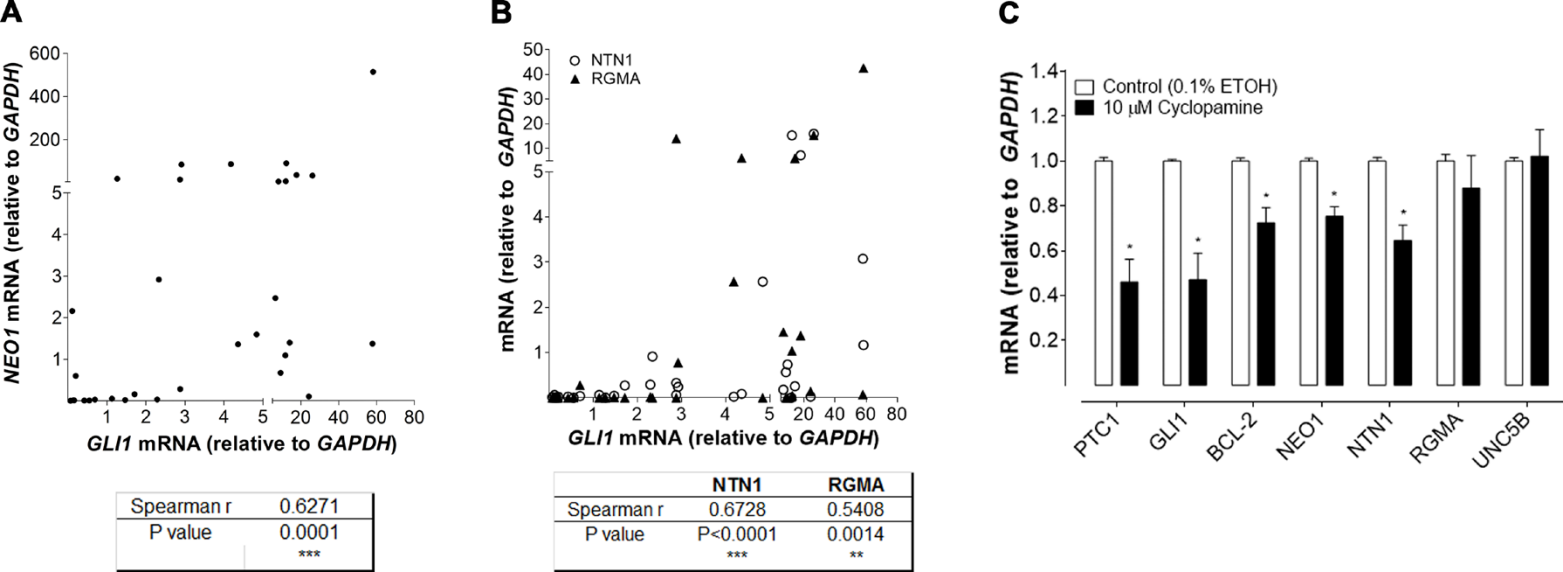
Positive correlation between NEO1 signaling and SHH/GLI pathway activation (**A**–**B**) mRNA levels of NEO1, GLI1, NTN1 and RGMA were quantified by qPCR in sporadic human BCC samples. (A) mRNA levels of *NEO1* have a positive and significant correlation with *GLI1* levels in human BCC samples (*n* = 32). Spearman r and *P* values were calculated and are shown in the table, ***p ≤ 0.001. (B) mRNA levels of NEO1 ligand: *NTN1*(○) and *RGMA*(▲) also correlate with *GLI1* levels. For each ligand, Spearman r and *P* values were calculated and are shown in the table, **p < 0.01; ***p ≤ 0.001. (**C**) Five human BCC samples were cultured as explants and treated with 10 μM cyclopamine or vehicle control (ETOH 0.1%) for 48 h. Cyclopamine treatment decreases the expression of downstream targets such as *GLI1*, *PTCH1*, *BCL2* and also *NEO1*. Of NEO1 ligands only *NTN1* expression was diminished after inhibitor treatment. Levels of *UNC5B*, another reported NTN1 receptor with no-known relationship to the SHH/GLI pathway is shown as control. Data are represented as mean ± SEM with * p < 0.05 according to Mann-Whitney test.

We expanded our analysis of NEO1 expression by performing quantitative analysis of *NEO1* mRNA levels in both human control skin and in a variety of sporadic BCC tumor samples (Figure 4A). We also quantified the mRNA levels of *GLI1*, and NEO1 ligands *NTN1* and *RGMA.* Control skin samples were obtained from non-related surgeries and there was no variation for any of the genes assessed according to excision site (Supplementary Figure 5A) or donor gender (Supplementary Figure 5B). Both *NEO1* and *GLI1* mRNA levels varied greatly among BCC samples (Figure 4A). In order to determine if there was a significant correlation between the level of *NEO1* transcript expression and the amplitude of SHH/GLI signaling pathway activity (using *GLI1* as a readout of pathway activation) we calculated the correlation Spearman r value between both genes. This analysis revealed that there was indeed a significant and positive correlation between *NEO1* and *GLI1* expression levels (i.e. as the level of *GLI1* increases the level of *NEO1* never decreases) (Figure 4A). NEO1 ligand transcript levels also varied greatly among BCC samples (Figure 4B). Both *NTN1* and *RGMA* were found to exhibit a significant positive correlation with *GLI1* (Figure 4B).

In order to address whether the correlation between *NEO1/NTN1/RGMA* and *GLI1* is directly attributable to these genes acting as downstream targets of SHH/GLI pathway activity, we performed *ex vivo* inhibition of human BCC explants. Consistent with effective pathway inhibition, we observed a decrease in *PTCH1*, *GLI1*, and *BCL2* mRNA levels (known SHH/GLI pathway target genes) upon treatment with the SMO antagonist, cyclopamine. In contrast to the mRNA level of *RGMA* or *UNC5B* (an unrelated NTN1 receptor), which did not change upon cyclopamine treatment, *NEO1* and *NTN1* displayed significant decreases in mRNA upon treatment (Figure 4C). These data demonstrate that transcriptional activation of both the *NEO1* receptor and *NTN1* ligand are modulated by SHH/GLI pathway activity.

### Neo1 expression and BCC progression

**Figure 5:**
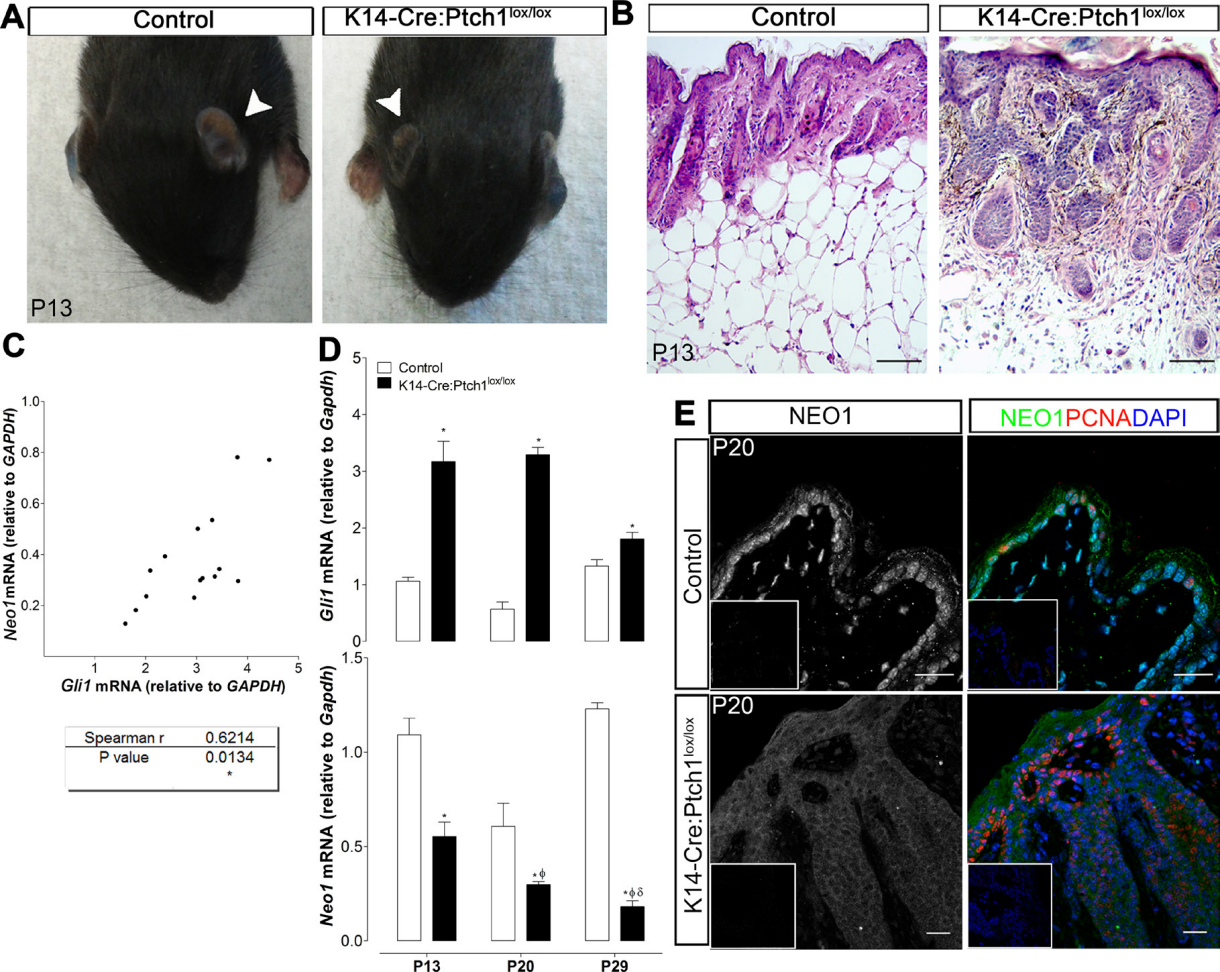
Neo1 decreases with BCC tumor progression (**A**) *K14-Cre:Ptch1*^lox/lox^ mouse generate skin lesions, being the phenotype at early ages mostly evident in ears (white arrows indicate ears of control and *K14-Cre:Ptch1*^lox/lox^ mice on P13) (**B**) H&E staining of ear skin showing micronodular BCC-like lesions in comparison to control skin on P13 mouse (bar = 100 μm). (**C**) mRNA levels were quantified by qPCR, showing a significant correlation between *Gli1* and *Neo1* levels on *K14-Cre:Ptch1*^lox/lox^ skin (*n* = 7). (**D**) *Gli1* mRNA levels of *K14-Cre:Ptc1*^lox/lox^ skin (*n* = 7) are upregulated in comparison to age-matched controls skin (*n* = 8) at postnatal days P13, P20 and P29. *Neo1* mRNA levels are downregulated in *K14-Cre:Ptch1*^lox/lox^ in comparison to age-matched controls skin. Levels of *Neo1* diminish significantly with tumor progression. Data is represented as mean ± SEM with *p* < 0.05 *vs*. age-matched control (*), *K14-Cre:Ptc1*^lox/lox^ P13 (ϕ*),* or *K14-Cre:Ptc1*^lox/lox^ P20 (δ); according to Mann-Whitney test. (**E**) NEO1 IF shows localization on basal cells of P20 control skin and dimmer staining on P20 K14-Cre:Ptch1^lox/lox^ skin, both on tumor nodules and epidermis. Proliferating tumor and basal cells are identified by PCNA staining (red). DAPI (blue) was used for nuclei staining (bar = 20 μm). Negative controls are shown as insets.

We next set out to address whether Neo1 levels remained static or varied during the course of BCC initiation and progression. In order to evaluate this, we made use of our previously published *K14-Cre:Ptch1*^*lox*/lox^ mouse model [26], whereby ablation of Ptch1 in mouse epidermal cells results in the rapid development of BCC-like lesions (Figure 5B). mRNA levels of *Neo1* and *Gli1* were quantified in skin samples obtained from control and *K14-Cre:Ptch1*^*lox*/lox^mice on P13, P20, and P29. In line with the results obtained from our human BCC analyses (Figure 4A), the level of *Neo1* and *Gli1* mRNA in *K14-Cre:Ptch1*^*lox*/lox^ skin presented with a significant positive correlation (Figure 5C). Consistent with Gli1 being upregulated in murine BCC, we observed high levels of *Gli1* mRNA in *K14-Cre:Ptch1*^*lox*/lox^ epidermis in all three BCC developmental stages assessed. In contrast, *Neo1* levels were significantly lower than control epidermis at all stages of BCC development (Figure 5D). Moreover, the level of *Neo1* decreased as BCC development progressed (lower levels at P29 than at P13) (Figure 5D). Immunofluorescence staining of Neo1 in control mouse skin revealed intense nuclear staining in the proliferative (PCNA positive) basal cell layer (Figure 5E). In contrast, Neo1 appeared dim and cytoplasmic within proliferative and non-proliferative regions of *K14-Cre:Ptch1*^*lox*/lox^ BCC (Figure 5E). These data suggest that in contrast to human BCC samples, whereby we observed high levels of NEO1 in mRNA and protein in a subset of BCC samples, murine BCC development is linked to a loss of *Neo1* expression.

### Association of NEO1 levels of expression with BCC aggressiveness

We performed a PCA analysis on the mRNA data of human BCC samples that included values of *NEO1*, its ligands, and the SHH/GLI pathway components, in order to assess the variation among the human sporadic BCC samples. This analysis revealed that most of the variance in the human BCC samples (87%) is due to the first component, and that almost all of the loading of this component was due to *NEO1* (Supplementary Figure 6).

**Figure 6:**
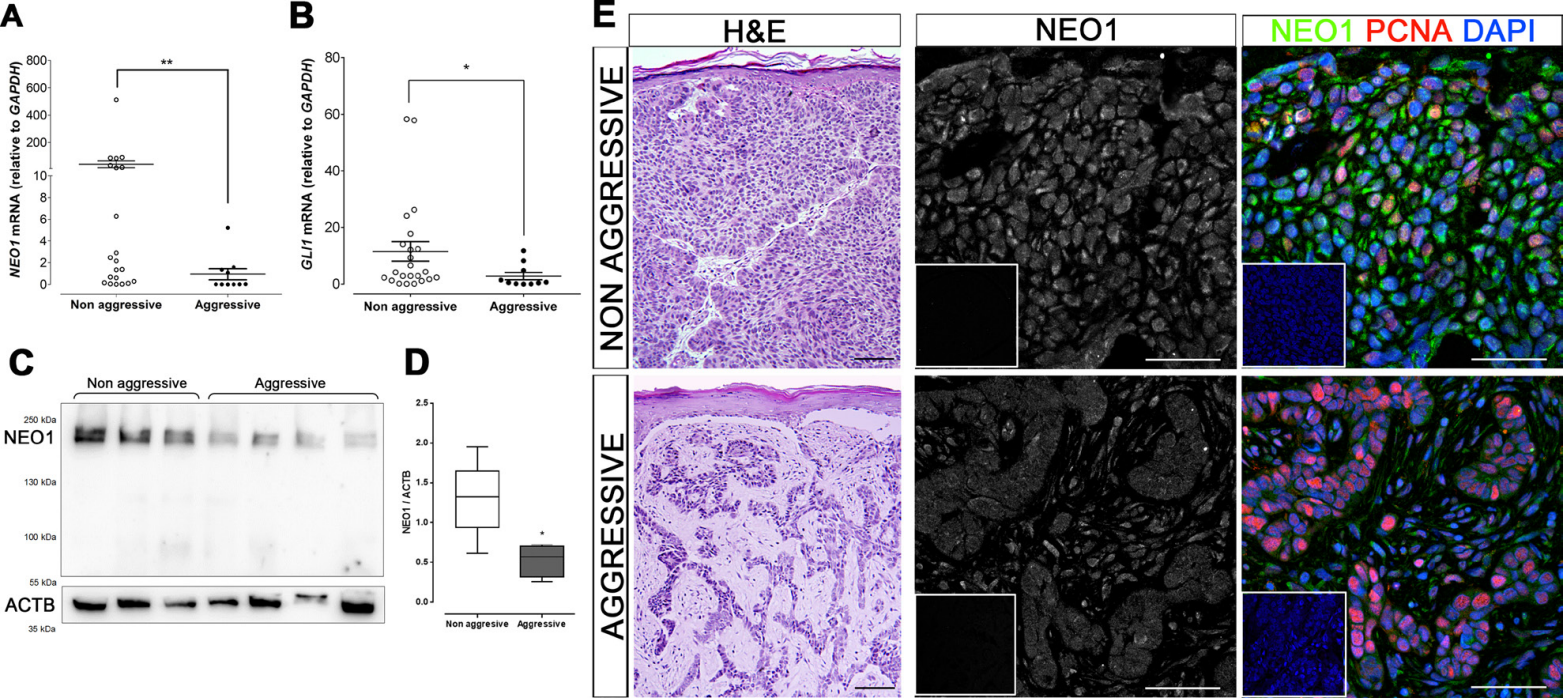
Low levels of NEO1 are related with BCC aggressiveness (**A**–**E**) Subtypes of sporadic human BCC samples were assessed by histomorphological analysis. mRNA levels of both *NEO1* (A) *and GLI1* (B) of aggressive BCC subtypes (*n* = 10) are significantly lower than levels of non-aggressive BCC (*n* = 22). Data is represented as mean ± SEM with *p < 0.05, **p< 0.01; according to Mann-Whitney test. (C) Representative WB membrane of NEO1 expression in non-aggressive and aggressive BCC subtypes, ACTB is shown as a loading control. (D) The levels of NEO1 in non-aggressive BCC is higher than in aggressive BCC subtypes. Data correspond to *n* = 8 for non-aggressive BCC samples and *n* = 4 for aggressive BCC samples and are normalized against ACTB expression. *p < 0.05, according to Mann-Whitney test. (E) H&E of representative BCC biopsies (left column, bar = 250 μm) show histological differences between aggressive and non-aggressive BCC subtypes. IF for NEO1 (green) and PCNA (red) of tumor nodules of these samples show strong staining on non-aggressive tumor cells and dimmer staining for aggressive tumor cells. DAPI (blue) was used for nuclei staining (bar = 50 μm). Negative controls are shown as insets.

With the assistance of anatomopathologists, all human sporadic BCC samples were classified according to their aggressive or non-aggressive histomorphological phenotype. We compared the expression levels of *NEO1* in these samples, and found that *NEO1* mRNA levels were significantly lower in aggressive human BCC samples than in non-aggressive samples (Figure 6A). In addition to this, due to the correlation between *NEO1* and *GLI1* expression and to the high variability of *GLI1* levels among human BCC samples (Figure 4A), we performed the same analysis on *GLI1* mRNA levels and found that they were also significantly lower in aggressive human BCC subtypes compared to non-aggressive subtype samples (Figure 6B). We corroborate this at a protein level by evaluating NEO1 abundance in a cohort of aggressive and non-aggressive sporadic BCC samples (Figure 6C). We found significant lower amounts of NEO1 in aggressive samples compared to non-aggressive ones (Figure 6D). Finally, we performed IF to verify NEO1 protein distribution in aggressive and non-aggressive human BCC samples. NEO1 presented with a strong cytoplasmic and nuclear expression profile in non-aggressive tumor tissues (Figure 6D), similar to the staining of NEO1 in basal cells of control mouse skin. In contrast, aggressive human BCC samples presented with dim and diffuse NEO1 expression, resembling the expression profile of mouse BCC samples (compare Figures 6D and 5E). These data reveal that lower levels of *NEO1* mRNA and NEO1 protein correlate with aggressive BCC subtypes.

## DISCUSSION

### NEO1 is expressed in epidermal basal cells

The SHH/GLI signaling pathway participates in the growth phase of the hair follicle cycle [9, 10]. Constitutive pathway activation has been linked to a wide variety of cancers, particularly BCC [11–13, 28–30]. The consequences of SHH/GLI pathway activation mostly relate to cell proliferation and control of the cell cycle [31]. In this study we assessed whether the DDR NEO1, which has been shown to be a transcriptional target gene of the SHH/GLI pathway [16], was expressed in the epidermis and if its expression correlated to the BCC aggressiveness behavior and progression.

Here we found that NEO1 is indeed expressed in the adult epidermis and that its expression is restricted to cells of the basal layer (Figure 1B), where SHH/GLI pathway components are also expressed [9]. Of note, a possible functional interaction between NEO1 and PTCH1, a classical SHH/GLI read-out gene, has been recently suggested [32]. The variations of *Neo1* mRNA levels in mouse skin, followed the same pattern as SHH/GLI pathway, assessed by the quantification of *Gli1* mRNA (Figure 2D). If *Neo1* levels vary along the length of the hair follicle remains to be determined.

NEO1 has many functions; it has been described to participate in neurogenesis during development acting both in proliferation and as a axonal guidance receptor and in adulthood by controlling migration and proliferation of neuroblast [17, 23, 33–36]. However, it is also implicated in the development and homeostasis of non-neural tissue such as that of the mammary gland, via its role in cell-cell adhesion [37] . Here, we present the expression of NEO1 basal cells of epidermis, but the mechanism and function of NEO1 in this context remains unknown. Our results lead us to propose a possible participation for NEO1 in skin homeostasis, in relation to the activity of the SHH-GLI pathway. It has been recently described that NEO1 participates in adherens junction formation and maintenance, which are fundamental for skin barrier function and control of basal cell polarity and therefore asymmetric cell division for stratification [38, 39]. NEO1 might be acting as an adhesive molecule in epidermal basal cells and therefore could be able to regulate both the proliferation and stemness of the cells [39, 40]. Nonetheless more research needs to be done in order to determine the precise function of NEO1 in skin formation and the hair follicle cycle.

### NEO1 is expressed in BCC but is downregulated in aggressive BCC subtypes and tumor progression

Importantly, NEO1 is also expressed in varying levels in BCCs. How the SHH/GLI pathway activation ultimately leads to a BCC phenotype is still relatively poorly understood. Previous work by other groups have shown variations in *GLI1* mRNA levels in BCC. Although they do not refer to this observation directly in the text, the data/figures indicate up to 100-fold variation [41–44]. In this study, we observe the same variation of *GLI1* levels (Figure 4A and 4B) and show that this variability could be correlated with the aggressiveness of the BCC tumor, where lower *GLI1* expression levels correspond to more aggressive BCC subtypes (Figure 6B). It has been reported that p53 is a negative regulator SHH/GLI1 pathway by inhibiting GLI1 activity [45]. P53 has mutations in almost 50% of sporadic BCCs where its downregulation increases SHH/GLI pathway activity leading to excessive proliferation and BCC development [46, 47]. Interestingly, higher p53 expression has been found in aggressive BCC subtypes, supporting our results that show low levels of GLI1 in aggressive BCC [46, 48]. Thus, p53 could be acting as a negative regulator of GLI1. Hence, we postulate that GLI1 activity may change during tumor progression and this could be related to acquisition of new mutations that regulate GLI1 activity. If this is the case, then it is possible that the SHH/GLI pathway may be essential for tumor onset and maintenance, but not for tumor invasion and aggressiveness.

*Neo1* appears to be downregulated in murine *Ptch1* mutant BCC and additionally, older *K14-Cre:Ptch1*^*lox*/lox^ mice with advanced BCC have lower *Neo1* expression (Figure 5D and 5E). We also show that NEO1 expression is significantly reduced in aggressive human BCC subtypes when compared to non-aggressive samples (Figure 6). This may be indicative that NEO1 is a negative marker for tumor aggressiveness and we would expect a decrease in its expression along tumor progression. Since NEO1 is related to cell-cell adhesion, its downregulation could promote epithelial-mesenchymal transition in the aggressive subtypes of BCC and explain the histology of this neoplasms where tumor cells form desegregated islands compared to big nodules of non-aggressive subtypes [4, 38, 49]. While NEO1 is significantly lower in aggressive BCC subtypes, we also detected low levels in specific non-aggressive BCC tumor samples. Here, the discretion lies in the fact that the samples used in this work are from a group of patients of varying ages, sunscreen use habits, and tumor stage and that research analysis was conducted in a single-blinded fashion. Therefore, the low NEO1 expression of the specific non-aggressive BCC samples may be as a result of patient-specific variables or due to the tumor's transition from a non-aggressive to an aggressive tumor. As NEO1 is expressed in both human and mouse normal basal cells, the decrease of NEO1 in BCC could be also explained by a loss of basal identity. Nonetheless, further analysis of *NEO1* variations in BCC subtypes is necessary in order to explain different tumor behaviors.

*NEO1* is not the only transcriptional target of SHH/GLI pathway that is downregulated in BCC. Research shows that *BCL-2*; a well-characterized transcriptional target of the SHH/GLI pathway [50] is also downregulated in aggressive BCC subtypes [51, 52]. Hence, although SHH/GLI signaling has been well characterized to play a pivotal role in driving BCC initiation, there is also evidence that further support our findings that SHH/GLI signaling activity is indeed down-regulated in aggressive subtypes and advanced stages of BCC. Here we show that the SHH/GLI pathway modulates not only *NEO1*, but also some of its ligands (Figure 4B and 4C). Taken together, the results reveal that SHH/GLI pathway activation or inactivation may be affecting the overall NEO1 signaling. The SHH/GLI pathway is in cross talk with several other signaling pathways. Besides SHH/GLI Wnt, Notch, and transforming growth factor β (TGF- β)/Bone Morphogenetic Protein (BMP) signaling are key among the pathways controlling epidermal lineage and homeostasis [53–55] and many other cancer related pathways are expressed distinctively among BCC subtypes [3]. NEO1 has recently been shown to function as a co- receptor for the BMP proteins that belong to the TGF- β superfamily, and RGMs signal via NEO1 and BMPs and physically connects both pathways [56]. NEO1 was also found to bind directly with BMP-2, BMP-4, BMP-6, and BMP-7 and to regulate negatively the functions of BMP [57]. Noteworthy, NEO1 may thus represent a critical node linking SHH/GLI and BMP signaling in BCC. It might therefore be important to simultaneously evaluate the respective contribution of both pathways on NEO1 function. Future research should take this into consideration and determine whether or not other pathways are involved in BCC aggressiveness and progression.

## MATERIALS AND METHODS

### Mice whole skin samples

Conditional basal cell-specific deletion of Ptch1 in mouse epidermis (*K14-Cre: Ptch1^lox/lox^*) was achieved by crossing C57 *Ptch1^lox/lox^* females with C57 *K14-Cre: Ptch1^lox/+^* males. All mice were genotyped on postnatal day 7 (P7). Littermates without the desired genotype (*K14-Cre: Ptch1^lox/lox^*) were used as controls.

Whole skin samples were obtained on P13, P20, and P29. These were either saved in RNAlater (Qiagen Hilden, Germany) for RNA extraction or fixed and paraffin embedded.

Mice were housed in a light-controlled facility at the Institute for Molecular Bioscience at the University of Queensland, Australia. All animal experimental protocols were reviewed and approved by the University of Queensland's Animal Ethics Committee.

### Human samples

Non-tumoral human skin was obtained from abdominoplasties and other non-related surgeries of 15 patients with the approval from the University of Chile's Clinical Hospital Ethical Committee and patient consent. Fat was removed and samples were subsequently saved in *RNAlater* (Qiagen Hilden, Germany) for RNA extraction, Lysis Buffuer for protein extraction or fixed and paraffin embedded.

BCC skin samples were obtained from 38 patients with prior approval from the Arturo Lopez Perez's Foundation Ethical Committee or the University of Chile's Clinical Ethical Committee and patient consent and patient consent. Samples were saved in *RNAlater* (Qiagen Hilden, Germany) for RNA or protein extraction. The foundation also supplied us with paraffin embedded histological slices from the same 32 patients.

Explant cultures were obtained from BCC skin samples of 5 patients with previous approval from the University of Chile's Clinical Ethical Committee and patient consent. These samples were immediately placed in DMEM culture medium (Gibco, Life Technologies) supplemented with antibiotics (100 U/ml Penicillin and 100μg/ml Streptomycin).

All human tumor samples used in this study were diagnosed, and morphologically typified, via routine histological analysis at the anatomopathology centers of both institutions.

In keeping with internal regulations and national requirements, all protocols were approved by the Bioethics Committees of both the Faculty of Science and the Faculty of Medicine of the University of Chile and the Bioethics Committee the National Fund for Science and Technology (FONDECYT).

### Cyclopamine treatment of *ex vivo* BCC samples

Fresh BCC samples were cultured as explants as described before [58] with some modifications. In brief, samples were sliced in 2 mm^3^ cubes, placed in culture inserts (pore size = 0.45 μm), partially dried for five minutes, and grown in 1 mL of DMEM culture medium supplemented with 20% FBS and antibiotics (100 U/ml Penicillin and 100μg/ml Streptomycin).

Each BCC explant was surgically divided in two: one was treated with the Hedgehog pathway inhibitor Cyclopamine (10 μM) diluted in 0.1% ethanol, and the other with the drug diluent. Explant cultures were collected two days post treatment, homogenized, and stored in RLT solution (Qiagen Hilden, Germany) for posterior RNA extraction.

### RNA extraction and reverse transcription

Mice RNA samples were prepared using Qlazol extraction (Qiagen Hilden, Germany) and purified with the QIAgen RNeasy Column Kit (Qiagen) with DNase digestion. cDNA was synthesized via the reverse transcription of 1 μg of RNA using the Superscript III Kit (Life Technologies, Grand Island, NY).

Human RNA samples were prepared using RNAsolv (Omega Bio-Tek) and treated with DNasa I kit (Invitrogen). The QIAgen RNeasy Fibrous Tissue Column Kit (Qiagen Hilden, Germany) was used for explant samples. cDNA was synthesized via the reverse transcription of 1 μg of RNA using the RevertAid Kit (LifeTechnologies). All cDNA samples were stored at –20°C.

### qPCR analysis

Mice cDNA samples were analyzed via quantitative PCR (qPCR) using Taqman probes from Life Technologies. Gapdh: 4352339E; Gli1: Mm00494654_m; Neo1: Mm00476326_m1; Ntn1: Mm00500896_m1; Rgma: Mm00624998_m1. Amplification was done on ViiA7 Real Time (Life Technologies).

Human cDNA samples were analyzed via qPCR using specifically designed primers as follows: GAPDH: Fw 5′-CAAGAAGGTGGTGAAGCAGGC-3′ and Rv 5′- CCACCACCCTGTTGCTGTAG-3′; PTCH1: Fw 5′- GGTGGAAGTTGGAGGACGAG-3′ and Rv 5′- CGCTTCTGTGGTCAGATTAG-3′; GLI1: Fw 5′- GGAGAAGCGTGAGCCTGAATC-3′ and Rv 5′- TGGATGTGCTCGCTGTTGATG-3′; NEO1: Fw 5′- GCTTCATCAAATTGACGTGGCGGA-3′ and Rv 5′- AGATGTACACGGTCGCTGGCATTA-3′; NTN1: Fw 5′- TGCAAGAAGGACTATGCCGTC-3′and Rv 5′- GCTCGTGCCCTGCTTATACAC-3′; RGMA: Fw 5′- ATGGATGGGTATGGGGAGAG-3′ and Rv 5′- TGCACTTGAGGATCTTGCAC-3′; BCL2: FW 5′- GAACTGGGGGAGGATTGTGG-3′ and Rv 5′- CCGTACAGTTCCACAAAGGC-3′; UNC5B: FW 5′-GGGCTGGAGGATTACTGGTG-3′ and RV 5′- TGCAGGAGAACCTCATGGTC-3′. Amplification was performed on Agilent Technologies Real Time Thermocycler.

### Histological analysis

Samples, both murine and human, were fixed for 12 to 24 hours with 4% PFA, dehydrated in solutions of increasing ethanol concentration, xylol, and subsequently embedded in paraffin. Parrafin embedded samples were cut into 7μm slices on a microtome (Leica).

Hematoxilin & Eosin (H&E) staining was performed for histological analysis, as described elsewhere (Villani et al., 2010). Samples where deparaffinated for one hour at 60°C, washed in xylol, and rehydrated in solutions of decreasing ethanol concentrations, incubated in Hematoxilin (Vector Laboratories, Burlingame CA) and Eosin Y (Sigma Aldrich, St Louis, MO), dehydrated in Xylol and mounted with Entellan.

### Immunostaining

Slices were treated as described for histological analysis. After rehydration, the antigens were retrieved by incubating the samples in a citrate solution (Vector). Tissues were blocked for one hour with a 1% BSA, 4% Horse Serum, and 0.02% Triton solution in PBS, and further incubated with the primary antibodies overnight at 4°C. The following primary antibodies were used: NEO1 H175 (Santa Cruz Biotechnology), PCNA 13-3900 (Invitrogen), RGMA AF2459 (R&D Systems), and NTN1 H-104 (Santa Cruz Biotechnology). They were subsequently incubated with the secondary antibodies for two hours at room temperature (25°C). The following secondary antibodies were used: biotinylated anti-rabbit/mouse IgG (Vector), biotinylated anti-goat Igg (Vector), and anti-mouse Alexa 488 (Invitrogen).

For Immunohistochemistry (IHC), slices were incubated with the Avidin–Biotinylated-HRP Complex (ABC Reagent, Vectastain) for 30 minutes and then revealed with 39-diaminobenzidine (ImmPACT DAB, Vector). Hematoxylin was used for nuclei staining. Slices were finally dehydrated and mounted with Entellan.

Immunofluorescence (IF) samples were incubated an extra hour with a streptavidin-A594 (Invitrogen). Dapi was used for nuclei staining. Slices were mounted with fluorescence mounting medium (Dako).

### Western blot

BCC samples were collected in Lysis Buffer lysis buffer containing protease inhibitor mix and mechanically homogenized. 60 μg of proteins were separated by SDS-PAGE and transferred onto nitrocellulose membranes. These were blocked for 1 hour in 5% milk at room temperature and then immune-bloted overnight with primary antibody goat anti-NEO1 (C-20 Santa Cruz Biotechnology) at 4°C. As loading control incubation with mouse anti-β Actin (Sigma-Aldrich, A5316) was performed for 1 hour at room temperature. Membranes were washed with TBS-T (TBS 0.1% Tween-20), and incubated with peroxidase linked anti-goat or anti-mouse (Jackson ImmunoResearch, 111-036-003; 115-036-003). Finally, antigens were detected by chemi- luminescence (Thermo Scientific).

### Microscopy and image analysis

Microphotographs of both H&Es and IHCs were taken with an optical microscope (Olympus BX51) equipped with a digital camera (Moticam 2500).

### Statistical analysis

All statistical analyses were completed using the Graphpad Prism V5.0 software. Normality was assessed with D’Agostino-Pearson test; calculation of correlation coefficient (r) of Spearman for variable correlation; and Mann-Whitney test for comparison between groups. All values are expressed as mean ± SEM. Numbers of independent sample donors or animals are indicated in each figure. *P* < 0.05 was considered as statistically significant. Principal Component Analysis (PCA) was performed using the Past v3.01 (Ø, Hammer and D.A.T. Harper) software.

## SUPPLEMENTARY MATERIALS AND FIGURES


