# Supplementary material for: Downregulation of the Sonic Hedgehog/Gli pathway transcriptional target Neogenin-1 is associated with basal cell carcinoma aggressiveness
Source: Oncotarget. 2017 Sep 19;8(48):84006–18. doi: 10.18632/oncotarget.21061 (PMC5663572; doi:10.18632/oncotarget.21061)
Supplement: Supplementary file 1 [file oncotarget-08-84006-s001.pdf]

Downregulation of the Sonic Hedgehog/Gli pathway transcriptional target Neogenin-1 is associated with basal cell carcinoma aggressiveness

SUPPLEMENTARY MATERIALS

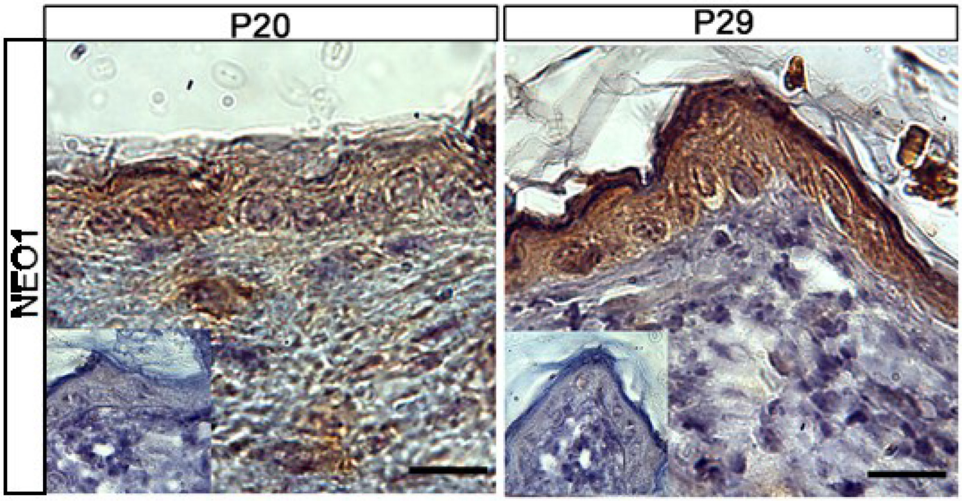

Supplementary Figure 1: Neo1 is expressed in mouse skin of different ages. IHC analysis of NEO1 in the basal cell population of mouse skin at P20 and P29. Images are representative photographs of *n* = 3 on P13 (bar = 25 µm). Negative controls are shown as insets.

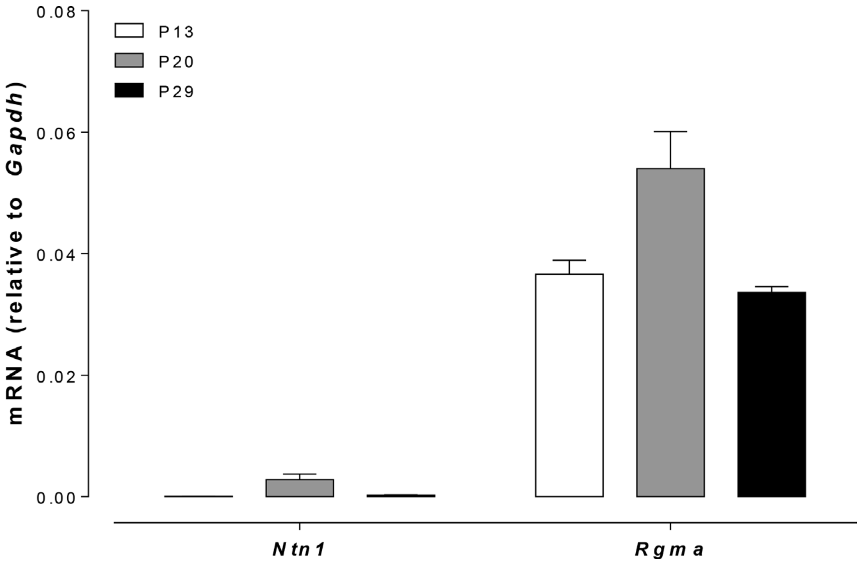

Supplementary Figure 2: Neo1 ligand expression varies during hair follicle cycle stages. Ntn1 and Rgma mRNA levels were quantified by qPCR on mouse skin (*n* = 8) at different ages. Data is shown as Mean ± SEM and values were normalized using Gapdh as housekeeping gene.

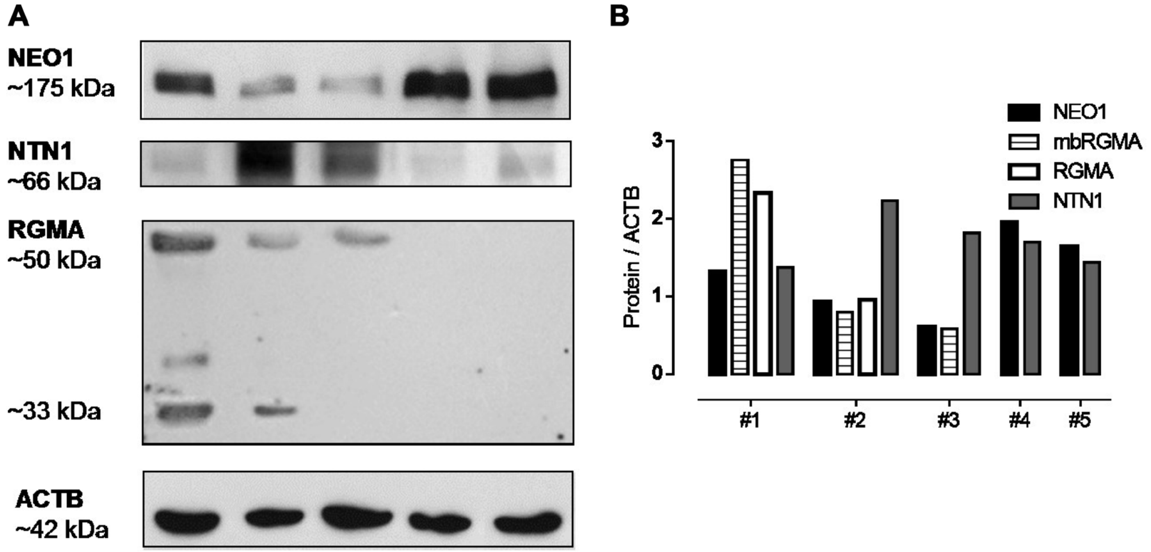

Supplementary Figure 3: NEO1 and its ligands are expressed in BCC. (**A**) NEO1, NTN1 and RGMA expression was assessed by WB in non-aggressive human sporadic BCC samples (*n* = 5), ACTB is shown as a loading control (**B**) Graph depicting the levels of NEO1, NTN1 and RGMA evaluated in (d) and normalized by ACTB expression.

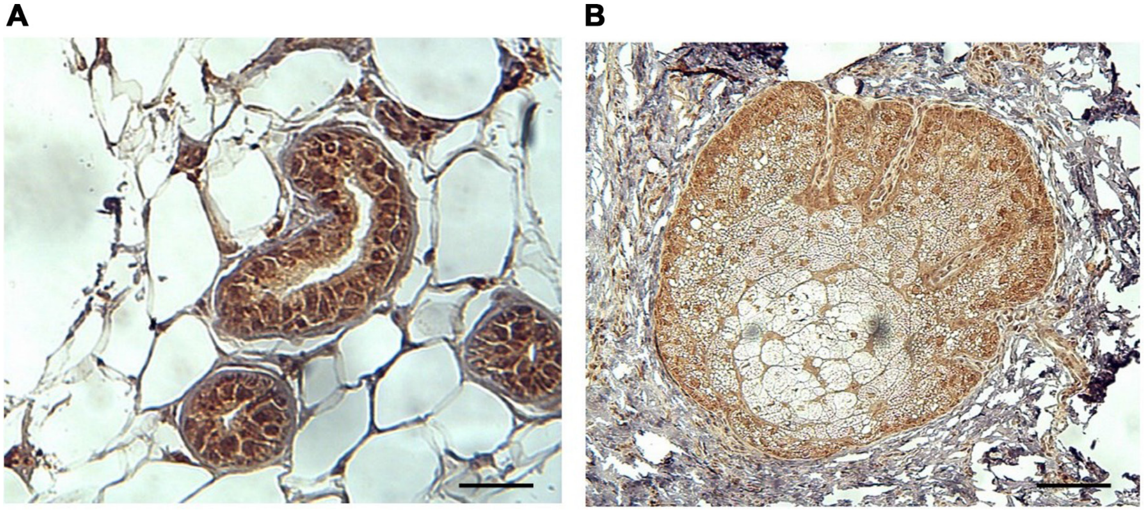

Supplementary Figure 4: NEO1 is expressed in skin appendages. IHQ analysis of normal skin dermis, showing NEO1 (brown staining) in both eccrine sweat gland (bar = 250 µm) (**A**) and sebaceal gland (bar = 500 µm) (**B**). Hematoxilin (blue) counterstain used to distinguish nuclei.

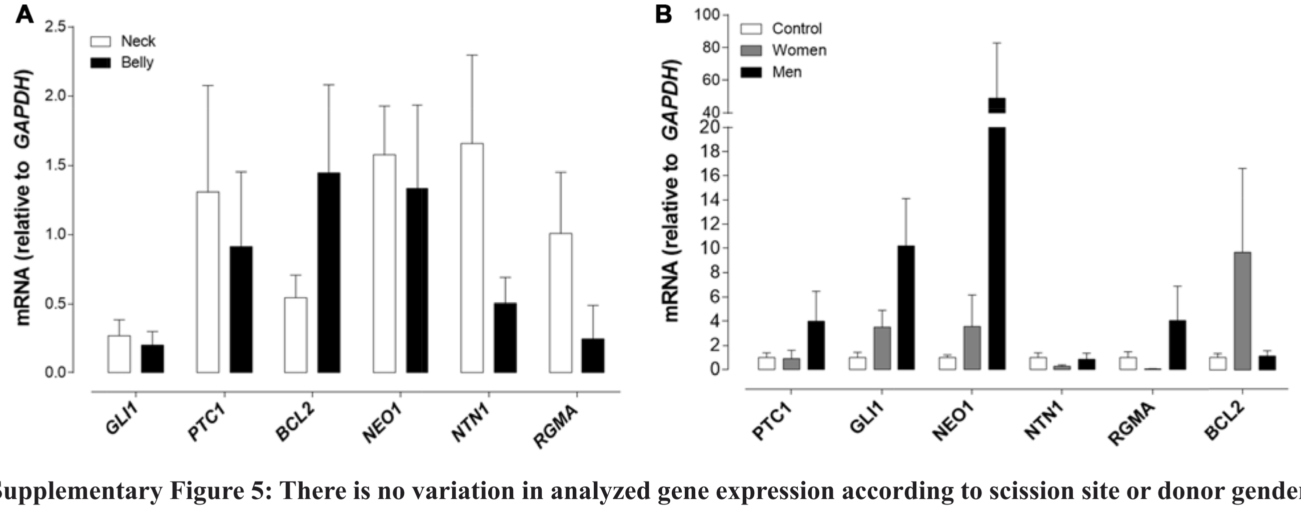

Supplementary Figure 5: There is no variation in analyzed gene expression according to scission site or donor gender. (**A**) mRNA quantification of all gene analyzed on healthy human skin shows no significant variation according to scission site of skin samples. (**B**) mRNA quantification of all indicated genes shows no significant difference according to donor gender of human BCC samples. Data is shown as Mean ± SEM and values were normalized using Gapdh as housekeeping gene.

PC	Eigenvalue	% variance
**1**	22732	87.073
**2**	3254.71	12.467
**3**	63.5507	0.24343
**4**	28.9076	0.11073

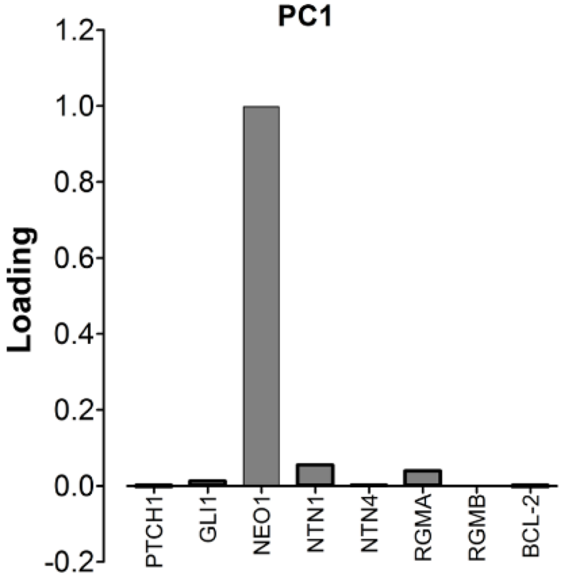

Supplementary Figure 6: Variance among sporadic human BCC samples is due to NEO1 expression. PCA of mRNA levels on different human BCC samples (*n* = 32), indicates that most of the variance is explained by NEO1 levels of expression.
